# Ventricular arrhythmias and conduction abnormalities following blunt cardiac injury

**DOI:** 10.1016/j.hroo.2025.02.012

**Published:** 2025-02-24

**Authors:** Mohammad Abed, Ali Lootah, Elizabeth Stephenson, Yuval Bitterman

**Affiliations:** Labatt Family Heart Centre, Department of Paediatrics, Hospital for Sick Children (SickKids), Toronto, Canada

**Keywords:** Blunt cardiac injury, RBBB, Trauma-related arrhythmia, Pediatric cardiac trauma, Ventricular arrhythmia

**What we learned from this case**

1. Blunt cardiac injury (BCI) in pediatrics can occur without multi-organ trauma or rib fractures and depends on projectile size and shape.
2. The most common BCI manifestations are RBBB, ST and T wave changes, and ventricular arrhythmias from the right ventricle.
3. Diagnosing and prognosticating BCI requires a high index of suspicion, testing, and monitoring, including troponin levels and ECG.

## Introduction

The presentation of blunt cardiac injury (BCI) is highly variable, ranging from asymptomatic elevation in biomarkers to life-threatening arrhythmias and sudden death.^1^

## Case report

A previously healthy 14-year-old boy presented to the emergency department after trauma to the left chest caused by a small metal carabiner that dislodged while exercising with rubber bands. He complained of severe local pain, dizziness, and shortness of breath that lasted 15 seconds. He did not endorse palpitations or experience syncope.

**Figure 1:**
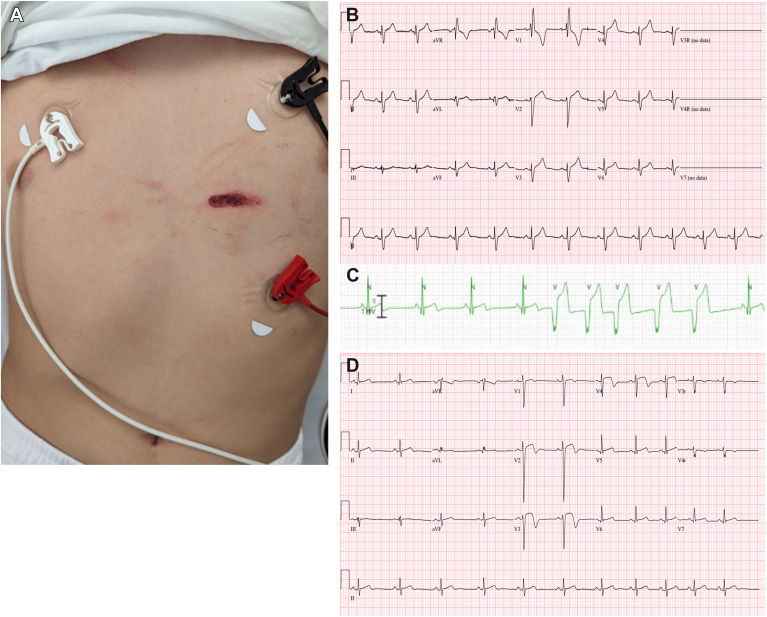
**A:** Picture of the chest wall trauma. **B:** ECG on arrival showing complete right bundle branch block (RBBB). **C:** Non-sustained run of accelerated idioventricular rhythm caught on telemetry. **D:** ECG performed 6 hours after the chest wall trauma showing resolution of the RBBB with residual ST changes.

Initial vital signs were normal. Physical examination revealed a 5-cm superficial abrasion between the 4th and 5th intercostal space on the left sternal border (Figure 1A).

An electrocardiogram (ECG) done 2 hours after the injury showed normal sinus rhythm with complete right bundle branch block (RBBB) (QRS duration, 118 milliseconds [Figure 1B]). The patient had no previous ECG for comparison. Chest radiograph was normal. Troponin I (TnI) levels were elevated (27,000 ng/L; upper limit of normal, <30.9 ng/L). Echocardiography demonstrated normal biventricular systolic function and no traumatic lesions.

Based on the concern for BCI, he was admitted to the cardiac critical care unit, where he later experienced nonsustained accelerated idioventricular rhythm and ventricular tachycardia at a maximum rate of 150 beats/min that were asymptomatic. These bouts had variable coupling intervals and were only captured on telemetry, precluding further assessment (Figure 1C).

An ECG done 6 hours post-BCI showed resolution of the RBBB and upsloping ST elevations in leads V2–V5 (Figure 1D). Repeated TnI measurements peaked at 6 hours post-BCI (32,970 ng/L) and decreased subsequently to 5,700 ng/L at discharge.

He remained admitted for 72 hours, with an unremarkable course. The accelerated idioventricular rhythm resolved by 48 hours. His predischarge ECG showed sinus bradycardia with improved ST elevations.

At follow-up, 3 weeks later, he remained asymptomatic. The ECG was unchanged.

A 24-hour Holter monitor demonstrated rare monomorphic single premature ventricular complexes (total of 43) and 4 monomorphic ventricular couplets (shortest coupling interval of 370 milliseconds) without higher-grade ectopy.

## Discussion

Herein we describe a case of relatively “minor” chest injury in an adolescent resulting in BCI. Our case highlights the dynamic nature of ECG changes and arrhythmias, and the importance of assessment for BCI.

The true incidence of BCI is unknown but estimated at 3% to 56% of adult BCI, depending on case definition and screening methods.^2^ Data for pediatric BCI are limited.^3^ In the largest pediatric case series, including 184 patients, BCI was associated with multi-organ trauma, but the authors concluded that incidence is probably higher because of increased chest wall compliance, and underestimated. Although BCI was found in only 0.2% of approximately 260,000 pediatric patients it carried a 25% all-cause mortality rate.^4^ These and other data led to the use of chest protective gear in certain sports.

The Eastern Association for the Surgery of Trauma recommends screening patients with suspected BCI with an ECG and TnI. If either is abnormal, admission for rhythm monitoring is recommended. If both are normal one can rule out BCI. However, in pediatrics, screening solely with TnI was associated with overdiagnosis.^5^

ECG changes in BCI vary, but ST segment changes are common in adults.^2^ The most common conduction abnormality after BCI is RBBB, with bifascicular blocks seen infrequently and almost no reports of isolated LBBB.^6^ This is probably attributable to the anatomic location of the right bundle and the right ventricle. Another factor is the timing with respect to the cardiac cycle: injuries during depolarization are more likely to cause bundle branch blocks, whereas injuries during repolarization may cause commotio cordis.^7^

BCI-associated complete atrioventricular block carried a 20% mortality rate and required pacemaker implantation in 50% of cases.^8^

BCI-induced arrhythmias are thought to be caused by mechanical-electrical coupling mediated by stretch-activated ion channels.^7^ Ventricular and atrial arrhythmias, including atrial fibrillation, have been reported.^2^ Imaging studies have correlated the origin of ventricular arrhythmias with trauma-related MRI or CT changes in the ventricular myocardium.^9^ Of note, BCI-associated arrhythmias can be delayed, underscoring the importance of observation for 48 to 72 hours.^9^^,^^10^ Our case presented with ventricular arrhythmia, with varying coupling interval hinting at an automatic source, which resolved within 48 hours.

Recent animal models have highlighted the importance of the trauma projectile shape. Hard, small, spherical objects had a higher likelihood of being arrhythmogenic, potentially because of greater energy transfer.^11^ The velocity of the projectile and location of trauma were also important. In our case, the relatively small solid object propelled by the band tension had hit a vulnerable spot in the chest.

## Conclusion

Diagnosing pediatric BCI is challenging, requiring a high index of suspicion even with relatively minor trauma. Symptoms range from mild ECG changes to severe arrhythmias.
